# Genotype–phenotype investigation of 35 patients from 11 unrelated families with camptodactyly–arthropathy–coxa vara–pericarditis (CACP) syndrome

**DOI:** 10.1002/mgg3.364

**Published:** 2018-02-04

**Authors:** Saliha Yilmaz, Dilek Uludağ Alkaya, Özgür Kasapçopur, Kenan Barut, Ekin S. Akdemir, Cemre Celen, Mark W. Youngblood, Katsuhito Yasuno, Kaya Bilguvar, Murat Günel, Beyhan Tüysüz

**Affiliations:** ^1^ Department of Neurosurgery Program on Neurogenetics Yale School of Medicine Yale University New Haven CT USA; ^2^ Department of Pediatric Genetics Cerrahpasa Medical Faculty Istanbul University Istanbul Turkey; ^3^ Department of Pediatric Rheumatology Cerrahpasa Medical Faculty Istanbul University Istanbul Turkey; ^4^ Department of Genetics Yale Center for Genome Analysis Yale School of Medicine New Haven CT USA

**Keywords:** camptodactyly–arthropathy–coxa vara–pericarditis, genotype–phenotype correlation, lubricin, NGS, noninflammatory arthropathy, nonsense‐mediated mRNA decay, *PRG4*

## Abstract

**Background:**

The camptodactyly–arthropathy–coxa vara–pericarditis syndrome (CACP) is a rare autosomal recessive condition characterized by camptodactyly, noninflammatory arthropathy, coxa vara, and pericarditis. CACP is caused by mutations in the proteoglycan 4 (*PRG4*) gene, which encodes a lubricating glycoprotein present in the synovial fluid and at the surface of articular cartilage.

**Methods:**

In the present study, we compared the clinical and molecular findings of CACP syndrome in 35 patients from 11 unrelated families. In 28 patients, whole exome sequencing was used to investigate genomic variations.

**Results:**

We found that camptodactyly of hands was the first symptom presented by most patients. Swelling of wrists, knees, and elbows began before 4 years of age, while the age of joint involvement was variable. Patients reported an increased pain level after the age of 10, and severe hip involvement developed after 20 years old. All patients presented developmental coxa vara and seven patients (~22%) had pleural effusion, pericarditis, and/or ascites. We identified nine novel genomic alterations, including the first case of homozygous complete deletion of exon 1 in the *PRG4* gene.

**Conclusion:**

With this study, we contribute to the catalog of CACP causing variants. We confirm that the skeletal component of this disease worsens with age, and presents the potential mechanisms for interfamily variability, by discussing the influence of a modifier gene and escape from nonsense‐mediated mRNA decay. We believe that this report will increase awareness of this familial arthropathic condition and the characteristic clinical and radiological findings will facilitate the differentiation from the common childhood rheumatic diseases such as juvenile idiopathic arthritis.

## 1 INTRODUCTION

The camptodactyly–arthropathy–coxa vara–pericarditis syndrome (CACP) is a rare autosomal recessive condition characterized by early onset camptodactyly, noninflammatory arthropathy with synovial hyperplasia, and progressive coxa vara deformity (MIM # 208250). Pericardial or pleural effusions have been observed in some patients (Faivre et al., 2000). In 1997, linkage studies on four consanguineous kindred with autosomal recessive CACP syndrome identified a common region of homozygosity among the affected individuals. The authors concluded that this shared interval of 1.9‐cM, which mapped to chromosome 1q25‐q31, contained the gene implicated in the disorder (Bahabri et al., 1998). A year later, Marcelino et al. (1999) used the same kindred to reduce the candidate interval to 2 Mb and identified four homozygous deletions in proteoglycan 4 (*PRG4*; OMIM: 604283). The *PRG4* gene, located on chr 1q25‐q31, contains 12 exons spanning 18 kb (Ikegawa, Sano, Koshizuka, & Nakamura, 2000). The product of this gene, lubricin, is the lubricating component in the final lubricating fraction of human synovial fluid. It has chondroprotective feature in synovial fluid and functions as boundary lubricant at the cartilage surface (Jay, Britt, & Cha, 2000). Since the molecular basis of CACP was revealed in 1999, seven additional studies have reported more than 13 CACP families with more than 22 unique *PRG4* deleterious mutations. All of these alterations are predicted to lead to a premature stop codon (PTC), except for one case (Marcelino et al., 1999). The syndrome presents a striking molecular homogeneity and a wide phenotypical heterogeneity (Faivre et al., 2000). During diagnosis, CACP syndrome may initially be easily confused with juvenile idiopathic arthritis (JIA), causing a delay in diagnosis and unnecessary treatment with antirheumatic drugs. With this study, we aimed to explore the detailed clinical and molecular data for 35 patients with CACP in 11 unrelated families in order to look for possible phenotype–genotype correlations and discuss for the intra‐ and interfamilial clinical variability reported in CACP population.

## 2 METHODS

### 2.1 Ethical Compliance

The study protocol was approved by the Yale Human Investigation Committee (protocol no. 0908005592). Written consents from all subjects were obtained by the referring physicians at participating institution.

### 2.2 Subjets and material

The study included 35 patients from 11 families who were clinically diagnosed with CACP within 15 years at the Pediatric Genetic Department of Istanbul University, Cerrahpaşa Medical Faculty.

The patients were diagnosed and followed by an experienced clinical genetics specialist. Written consent for permission to participate in molecular studies and permission for photographs were obtained from the families by clinical genetics specialist. All the families have been selected retrospectively. The majority of patients were first admitted to pediatric rheumatology department due to arthritis complaint, after that they were referred to clinical genetics unit from the pediatric rheumatology department for the diagnosis of skeletal dysplasia. Ten of the families presented in this study are from a southeast region of Turkey where rates of consanguineous marriage are high. Family 10 is from the north region of Iraq. Nine of the 11 families are reported to be consanguineous (see Table 1). Blood samples were collected from patients and their parents (Table 1). Of the total of 35 subjects with available phenotypic data, 28 of them had blood samples collected. Genomic DNA was extracted from peripheral blood using PureGene DNA isolation kit (Gentra Systems, Minneapolis, MN) according to the manufacturer's instructions.

**Table 1:** Clinical and radiological features of 35 patients from eleven families with CACP

Family Number		Family 1 (5 families from the same clan)						Family 2			Family 3	Family 4	Family 5		Family 6	Family 7		Family 8					Family 9				Family 10	Family 11			Total
Number of children		38 subjects from 5 families									3	1	3		3	4		6 in one family and NA In cousin's family					15 subject in 3 families				2	4			
Number of affected children		7M/5F						3			1	1	2		1	2		3 Males in one family+ 1F,1M cousin					4 Male (+53 years old uncle with similar findings),1 F				1F	3			
Number of Male/Female		NA						3M			1M/2F	1M	1M/2F		1M/2F	1M/3F		2F/5M in one family, 4F and 4Males in cousin's family					6M/9F				1M/1F	2F/1M			
Patient number		1	2	3	4	5	6‐12	13	14	15	16	17	18	19	20	21	22	23	24	25	26	27	28	29	30	31	32	33	34	35	NA
PatientID		NG1620‐1	NG1620‐11	NG1620‐2	NG2222‐1	NG1620‐3		NG1848‐5	NG1848‐2	NG1848‐1	NG1850‐1	NG2147‐1	NG2619‐1	NG2619‐2	NG2620‐1	NG1849‐1	NG1849‐2	NG2630‐1	NA	NA	NA	NA	NG2798‐1	NG2798‐2	NG2798‐5	NG2798‐4	NG2966‐1	NG3130‐1	NG3130‐2	NG3130‐3	NA
Screening method		Exome	Sanger	Sanger	Sanger	Sanger	Sanger	Sanger	Sanger	Sanger	Exome	Sanger	Exome	Exome	Exome	Exome	Exome	Exome	Not screened	Not screened	Not screened	Not screened	Exome	Sanger	Sanger	Exome	Sanger	Sanger	Sanger	Sanger	NA
Current Age (years)		13	6	20	*8*	41	6‐32 yrs	32	30	24	17 1/2	14	9 1/2	4 1/2	15	22	11	16	14	12	25	21	3 1/2	16 1/2	24	53	5	18	15	11	3.5 to 53
Gender		F	M	M	M	M	3M/4F	M	M	M	F	M	F	F	F	F	M	M	M	M	F	M	M	M	M	F	F	F	F	M	17M/13F
Parental Consanguinity		The same clan						First cousin			Secound cousin	Secound cousin	First cousin		First cousin	Geographic proximity (Close villages)		First cousin					The same clan				First cousin	Same village			8/10
Age at Onset		5‐6 mo	1 yr	NA*	1 mo	1 yr	NA	2 yrs	2 yrs	2 yrs	1y	7mo	1 yr	1 yr	2 yrs	1 yr	1 yr	2‐3					9‐10 mo	NA	NA	NA	2	2y	3y	1.5y	1‐24 mo
First findings		C	C	C	W	C	NA	C	C	C	C	C	C	C	C	Knee	C	Knee	Knee	Knee	Knee	Knee	C	C	C	C	W	C	Knee	C	19C, 2W, 7K
Initial diagnosis		JIA	‐	JIA	‐	JIA	‐	JIA	JIA	‐	JIA	JIA	JIA	‐	JIA	JIA	‐	JIA	‐	‐	JIA	‐	‐	JIA	JIA	JIA	‐	JIA	‐	‐	16JIA
Age at diagnosis		5	1	10	3	34	6‐32 yr	18	16	10	12.5	1	6.5y	15mo	13.5	15	3	13	11	9	22	18	2	17	24	52	3 1/2	18	15	24	1‐52 yrs
Selected Clinical features for PhenoScore																															
Camptodactyly Hands/feet	*1*	+	+	+	+	+	7/7	+	+	+	+	+	+	+	+	+	+	+	+	+	+	+	+	+	+	+	+	+	+	+	35/35
Arthropathy																															
Wrists	***2***	+	+	+	+	+	7/7	+	+	+	+	+	+	+	+	+	+	+	+	+	+	+	+	+	+	+	+	+	+	+	35/35
Elbows	***3***	+	+	+	+	+	7/7	+	+	+	+	+	+	+	+	+	+	+	+	+	+	+	+	+	+	+	+	+	+	+	35/35
Knees	***4***	+	+	+	+	+	7/7	+	+	+	+	+	+	+	+	+	+	+	+	+	+	+	+	+	+	+	+	+	+	+	35/35
Hip	***5***	‐	‐	+	+	+	7/7	+	+	+	+	+	+	‐	+	‐	‐	+	‐	‐	+	+	‐	+	+	+	‐	+	+	+	26/35
Ankles	***6***	+	‐	‐	‐	+	NA	+	+	+	+	+	+	‐	+	+	+	+	‐	‐	+	+	‐	+	+	+	‐	+	+	+	20/28
Radiological Findings																															
Coxa vara	***7***	+	+	+	+	+	7/7	+	+	+	+	+	+	+	+	+	+	+	+	+	+	+	+	+	+	+	+	+	+	+	35/35
Flattened femoral heads	***8***	+	+	+	+	+	7/7	+	+	+	+	+	+	+	+	+	+	+	+	+	+	+	+	+	+	+	+	+	+	+	35/35
Short femoral neck	***9***	+	+	+	+	+	7/7	+	+	+	+	+	‐	‐	‐	+	‐	+	+	+	+	+	+	+	+	+	+	+	+	+	22/35
Osteoporosis	***10***	+	‐	+	‐	+	7/7	+	+	+	+	‐	+	‐	+	+	‐	+	‐	+	‐	+	‐	+	+	+	+	‐	+	+	26/35
Intra‐osseos cysts	***11***	‐	‐	+	‐	+	7/7	‐	‐	‐	+	+	‐	‐	+	‐	‐	‐	‐	‐	‐	+	‐	‐	‐	‐	‐	‐	‐	‐	13/35
Increased lumbar lordosis	***12***	+	‐	+	+	+	7/7	+	+	+	+	+	+	‐	+	+	+	+	+	+	+	+	‐	+	+	+	‐	+	+	+	31/35
Pain	***13***	+	‐	+ Hip	‐	+ Hip	NA	+	+	+ wrist	+	‐	‐	‐	+	‐	‐	+	+	‐	+	+	‐	‐	+	+	‐	‐	‐	‐	14/28
Surgery	***14***	‐	‐	‐	‐	+ Hip	NA	‐	+ Knee	+ wrist	‐	‐	‐	‐*	‐	+ hand	‐	‐	‐	‐	‐	‐	‐	‐	Sol 2 toe	Left elbow	‐	‐	‐	‐	6/28
Pericar/Acid/Pleur	***17***	‐/‐/‐	‐/‐/‐	‐/‐/‐	‐/‐/‐	‐/‐/+	1 (Perikardit)/7	‐/‐/‐	‐/‐/+	+/‐/‐	+/+/+	‐/‐/‐	‐/‐/‐	‐/‐/‐	‐/‐/‐	‐/‐/‐	‐/‐/‐	‐/‐/‐	‐/‐/‐	‐/‐/‐	‐/‐/‐	‐/‐/‐	‐/‐/‐	‐/‐/‐	‐/‐/‐	/‐/‐	/‐/‐/‐	‐/‐/‐	‐/‐/‐	‐/‐/‐	3Per, 1Acid, 3Ple
Echo	***19***	MVP MR	N	MVP MR	N	N	NA	N	N	Pericar	Pericar	MR	N	N	N	N	MVP MR	N	N	N	NA	NA	N	N	N	N	N	N	N	N	4MR, 3MVP, 1Peri
PhenoScore																															
Number of positif clinical feature out of 14	11	7	12	9	14		12	13	13	13	11	10	6	12	10	8	12	9	9	11	13	7	11	13	13	8	10	11	11		
Phenoscore (100% = 14 clinical feature present)	78.57	50.00	85.71	64.29	100.00		85.71	92.86	92.86	92.86	78.57	71.43	42.86	85.71	71.43	57.14	85.71	64.29	64.29	78.57	92.86	50.00	78.57	92.86	92.86	57.14	71.43	78.57	78.57		

C, Camptodactyly; W, Wrist; K, Knee; JIA, Juvenile idiopatic artritis; MVP, mitral valve prolapsus; MR, Mitral Regurtitation; mo, month; N, Normal; NA, Not available; Pericar, Pericarditis; Pleur, Pleuritis; yr, year.

### 2.3 Whole exome sequencing and analysis

For each family, genomics DNA from the index case was selected for whole exome sequencing (Table 1). One microgram of DNA was processed at the Yale Center for Genome Analysis (YCGA). Exome capture was performed using the NimbleGen 2.1 M human exome array (Roche Nimblegen, Inc., Madison, WI, USA) according to the manufacturer's protocol along with modifications previously described in the literature (Bilguvar et al., 2010; Clark et al., 2013). Exome library sequencing was performed using an Illumina HiSeq2000 with barcoding technology, paired end analysis, and six samples per lane. Variants were filtered and annotated with an in‐house bioinformatic pipeline devised by our research team (Caglayan et al., 2016; Clark et al., 2013). We analyzed the sequence reads of length 74 bp that passed the quality filter in the CASAVA pipeline (Illumina, Inc.). We use the gatkExome.rms pipeline as described at http://campuspress.yale.edu/knightlab/ruddle/gatkexome/. A detailed description of the software and pipeline can be found at http://campuspress.yale.edu/knightlab/. Reads were processed according to the GATK “best practices” pipeline for alignment and joint calling. Variants falling in genes previously associated with skeletal manifestations were annotated as such, based on occurrence in a catalog of 903 genes from the Online Mendelian Inheritance in Man (OMIM) database (as per march 2016; see Appendix S5; online Mendelian Inheritance in Man, OMIM^®^; McKusick‐Nathans Institute of Genetic Medicine, Johns Hopkins University [Baltimore, MD]; https://omim.org/). In other words, this list represents genes leading to skeletal manifestations when mutated and therefore potentially implicated in our patient's phenotype. *PRG4* gene is included in this list. We prioritized the list of variants for each index cases according to the (a) homozygous/heterozygous status, (b) occurrence in the list of 903 OMIM genes (Appendix S5), (c) the deleterious nature of the variant (as described above), and (d) the minor allele frequency. We used Sanger sequencing to confirm candidate variants. After identification of *PRG4* gene mutations, we tracked the segregation of these variants among available family members. We also searched for deleterious mutations in genes known to be connected to *PRG4* gene or its pathway.

### 2.4 Sanger sequencing

Exome results were evaluated by Sanger sequencing using KAPA HiFi HotStart Ready Mix PCR Kit (Kapa Biosystems) and the standard manufacturer's protocols. A difficult portion of exon 6 (Exon 6_2) contained imperfect repeats, and 500–800 ng of genomics DNA was alternatively amplified using AmpliTaq Gold^®^ DNA Polymerase (Applied Biosystems Inc.) with final concentration of 1.5 mm MgCl_2_ (Appendix S4). Amplicons were generated using ABI 9800 Fast Thermo cyclers (Applied Biosystems, Foster City, CA, USA), and post cycle sequencing, clean‐up was carried out with the CleanSEQ System (Beckman Coulter Genomics, Danvers, MA, USA). The amplicons were analyzed on 3730xl DNA Analyzer (Applied Biosystems Inc.). We used the following GenBank reference sequences for *PRG4* gene: genomic reference, NG_008248.1; transcript reference, NM_005807.3.

### 2.5 *PRG4* genomic DNA quantification by qPCR

We screened for *PRG4* loss with quantitative real‐time PCR (Q‐PCR) using Fast SYBR^®^ Green Master Mix (Roche Applied Science, Indianapolis, IN, USA). For each sample, six pairs of primers that span the *PRG4* gene were used for quantification, with subsequent normalization using primers on chromosomes 11 and 16 (Appendix S1). Samples and controls were run in triplicate. Dissociation curves were generated to ensure primer specificity. For each primer pair, we evaluated the PCR efficiency with a dilution series of a reference DNA (Promega, Madison, WI, USA) and found an *R* ^2^ > .99 for all cases. To determine the threshold cycle, female reference DNA (Promega, Madison, WI, USA), diluted at 21 ng/μl, was used for four serial dilutions from 1/4 to 1/256 fold. We considered a ratio ≤0.7 as loss and a ratio ≥1.3 as a gain. Each Q‐PCR run also included commercially available reference female DNA. We used CFX Manager™ software for data analysis (Bio‐Rad, CA, USA).

### 2.6 Statistics

To assess the clinical severity of each case, we defined a nonweighted “Phenoscore” based on 14 skeletal clinical features: camptodactyly of hands/feet (1), arthropathy of wrists (2), arthropathy of elbows (3), arthropathy of knees (4), arthropathy of hip (5), arthropathy of ankles (6), coxa vara (7), flattened femoral heads (8), short femoral neck (9), osteoporosis (10), intraosseous cysts (11), increased lumbar lordosis (12), pain (13), and surgery (14). The presence of a feature is represented by “+” and the absence by “−”. We count all “+” to attribute a numerical score to each individual patient. Therefore, the Phenoscore represent the presence of these features and not a degree of severity of each feature. A patient presenting all 14 skeletal clinical features had a Phenoscore of 100%. We used software GraphPad Prism 7 for statistical analyses and Spearman's rank‐order correlation coefficient to measure the strength of association between age and Phenoscore. The binominal test was used to determine gender bias compared to the theoretical ratio of 1:1, as well as mutations distribution. We used Fisher's exact test to look for link between gender and outcome (CACP vs. non‐CACP).

### 2.7 Database submission

All novel validated *PRG4* mutations have been submitted to the Locus Specific Mutation Database and Leiden Open Variation Database (LOVD).

## 3 RESULTS

### 3.1 Patients

**Figure 1:**
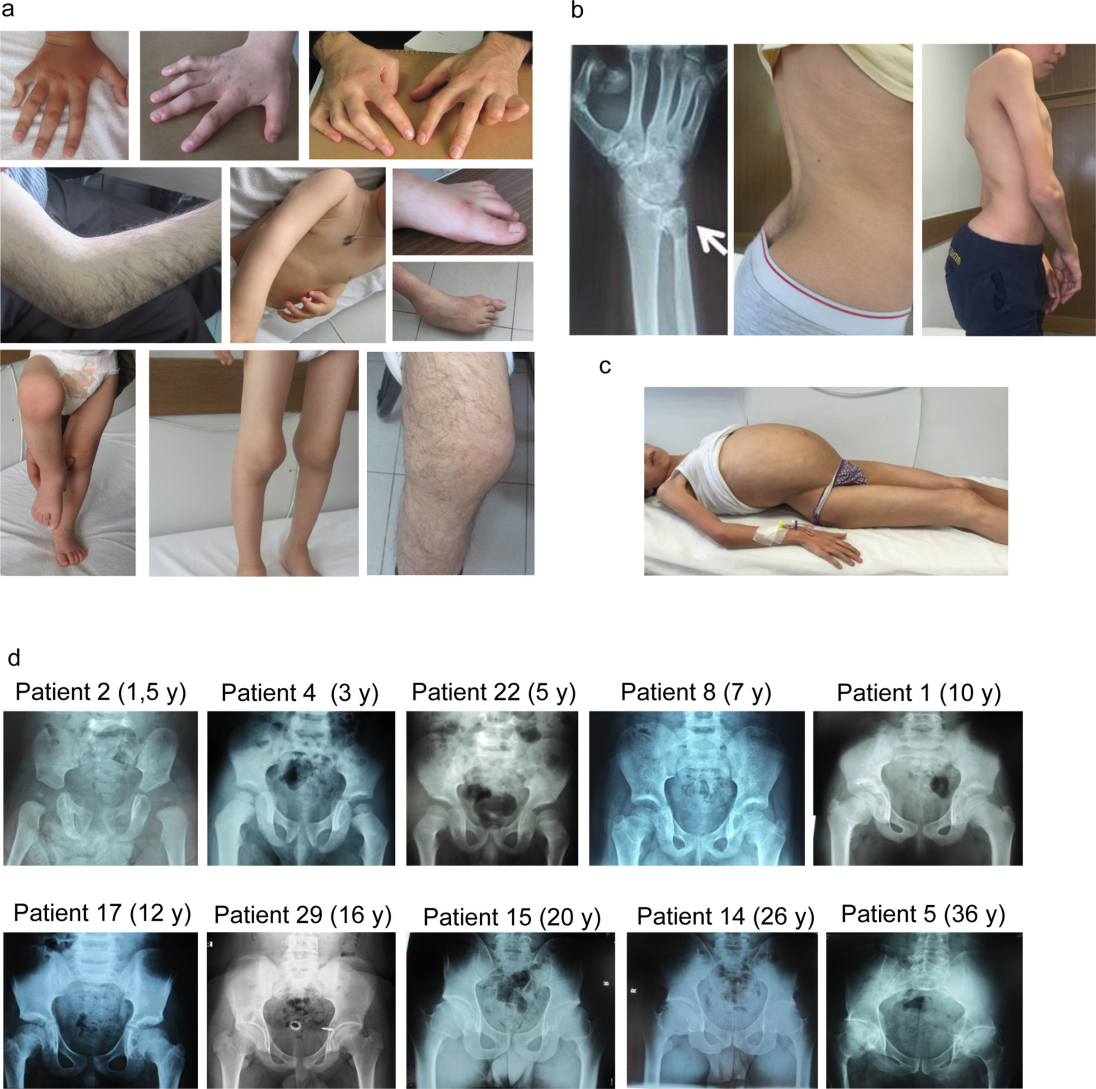
(a) Variable degree of camptodactyly and large joints involvement of the patients at different ages. (b) Cystic radiolucent lesion on wrist and variable degrees of lumbar lordosis. (c) Ascites in patient 16 from family 3. (d) Hand X‐ray of patient 5 revealed cystic radiolucent lesion on distal metaphysis of ulna. (e) Pelvis imaging of the patients at different ages showed narrowing acetabular space and irregularity of femoral capitis with aging, osteoporosis, short femoral neck, and mild–moderate coxa vara

Thirty‐five patients were included in this study, with a median age of 16 (3.5–53 years) and mean follow‐up duration of 7.8 years (0.5–16 years). Consanguinity was reported in 9 of the 11 unrelated families. Seven families had more than one affected subject. The age at diagnosis ranged from 1 to 52 years old. The clinical data of affected individuals are provided in Table 1. Camptodactyly was the first finding in 68% of patients (19 of the 28) (Table 1 and Figure 1a). In most patients, the age of onset for camptodactyly was approximately 1‐year‐old, while the swellings of the wrists, knees, and elbows began around the age of 4 (wrists being the first joints affected). Large joint involvement varied among patients. Older patients reported increases in pain level after the age of 10, corresponding to an increase in large joint contractures.

Severe hip and vertebral involvement were developed after 20 years of age. Seven patients had pleural effusion, ascites, and/or pericarditis. Four patients had mitral regurgitation or mitral valve prolapse on echocardiography. Abnormal skeletal radiographies included osteoporosis, enlarged flat femoral head with short femoral neck, small iliac wings enlargement of joint spaces, and mild–moderate coxa vara in all the patients. We also found cystic radiolucent lesion on wrist X‐ray in some patients (Figure 1b).

### 3.2 Molecular studies

**Figure 2:**
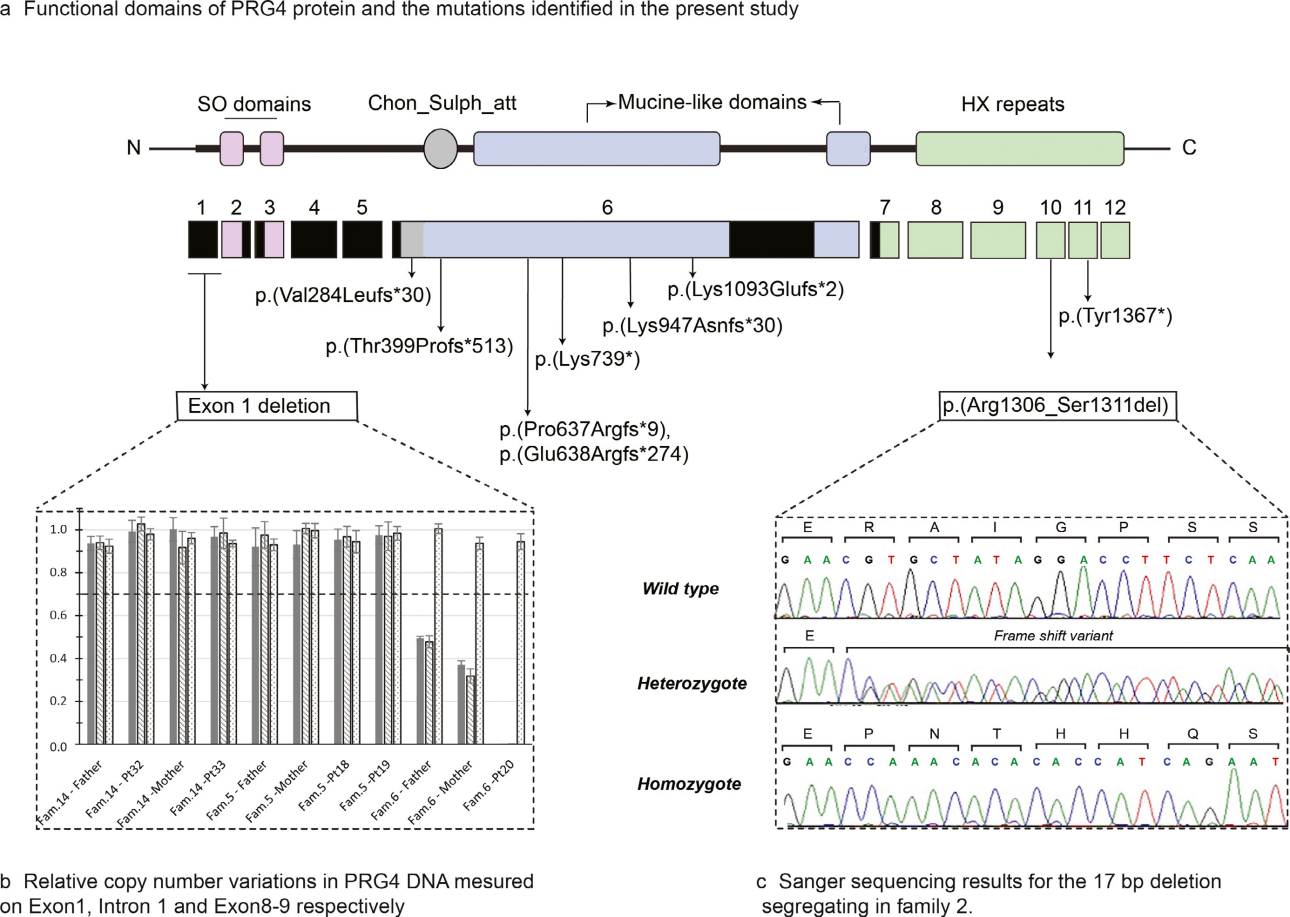
Structure of PRG4 protein and mutations identified in our cohort. (a) Functional domains of PRG4 protein and the mutations identified in the present study. SO domains, somatomedin B‐like domains; HX repeats, hemopexin‐like repeats; Chon_Sulph_att : chondroitin sulfate attachment site. (b) The graph represents the relative normalized copy number variation of *PRG4* DNA for primer pairs Ex1 (located on exon 1), E1I1 (located between exon 1 and 2) and Ex8–9 (located between exon 8 and 9), respectively. Patient 20 from family 6 presents a homozygous deletion of exon 1 detected by primer pairs Ex1 and Ex1I1, while primer Ex8–9 shows no variation in copy number. The parents of patient 20 are heterozygous for the deletion identified in patient 20. Families 5 and 14 do not show any copy number variation. (c) Sanger sequencing results for the 17‐bp deletion in exon 10 (p.1306fs) segregating in family 2. The affected three siblings all present the 17‐bp deletion (homozygous profile), both parents carry one copy of the deletion (heterozygous profile) and the control DNA show two copies of the wild‐type sequence

Whole exome analysis of family 1–subject 1 revealed a homozygous deletion in exon 6 of the *PRG4* gene. *PRG4* is known to be implicated in CACP, and all five affected cases carried the homozygous deletion while the parents carried the heterozygous variant (Figure 2c). We also carried out Sanger sequencing of the DNA of seven nonaffected individuals from family 1 and found that six subjects were heterozygous for the variant while one subject carried the homozygous wild‐type allele (data not shown). Expending our analysis to other families, we identified nine unique deleterious mutations in *PRG4* among 11 unrelated families (Figure 2a and Table 2). Families 3 and 7 harbored compound heterozygous mutations, while the remaining families were homozygous for the identified mutation. Particularly, we report the 17 bp homozygous deletions (c.3918delGTGCTATAGGACCTTCT) in exon 10 (family 2) as well as the homozygous deletion of exon 1 (family 6). This is the first report of deletion of a complete *PRG4* exon in CACP. The homozygous deletion of exon 1 was validated by quantitative PCR. In another patient (patient 16 family 3), we found a deletion in exon 1 based on Q‐PCR, but did not see any gain or loss on the other tested regions of *PRG4* gene (Figure 2b). In concordance with this observation, the parent's DNA showed a heterozygous loss of exon 1 while the probes pairs designed on other parts of *PRG4* DNA and the control primers did not show any copy number variation (CNV) (Figure 2b). We also screened other families but did not identify additional losses (data not shown).

**Table 2:** Mutations identified in the *PRG4* gene

Family number	Family ID	Exon	Mutation status	(hg19)Genomic DNA change on chromosome 1	DNA change NM_005807.3	Predicted protein change
1	NG1620	6	Homozygous	g.186276045delC	c.1194delC	p.(Thr399Profs*513)
2	NG1848	10	Homozygous	g.186281430_186281447del	c.3917_3934del	p.(Arg1306_Ser1311del)
3	NG1850	11;6	Compound heterozygous	g.186278127_186278128delAA; g.186282010C>G	c.3276_3277delAA; c.4101C>G	p.(Lys1093Glufs*2); p.(Tyr1367*)
4	NG2147	6	Homozygous	g.186278127_186278128delAA	c.3276_3277delAA	p.(Lys1093Glufs*2)
5	NG2619	6	Homozygous	g.186276043delC	c.1192delC	p.(Thr399Profs*513)
6	NG2620	1	Homozygous	g.(?_186265850)_(186266785_?)		
7	NG1849	6;6	Compound heterozygous	g.186276762delT; g.186277066A>T	c.1911delT; c.2215A>T	p.(Glu638Argfs*274); p.(Lys739*)
8	NG2630	6	Homozygous	g.186276761_186276762delCT	c.1910_1911delCT	p.(Pro637Argfs*9)
9	NG2798	6	Homozygous	g.186277688_186277689delAA	c.2837_2838delAA	p.(Lys947Asnfs*30)
10	NG2966	6	Homozygous	g.186275700delA	c.849delA	p.(Val284Leufs*30)
11	NG3130	11	Homozygous	g.186282010C>G	c.4101C>G	p.(Tyr1367*)

**Figure 3:**
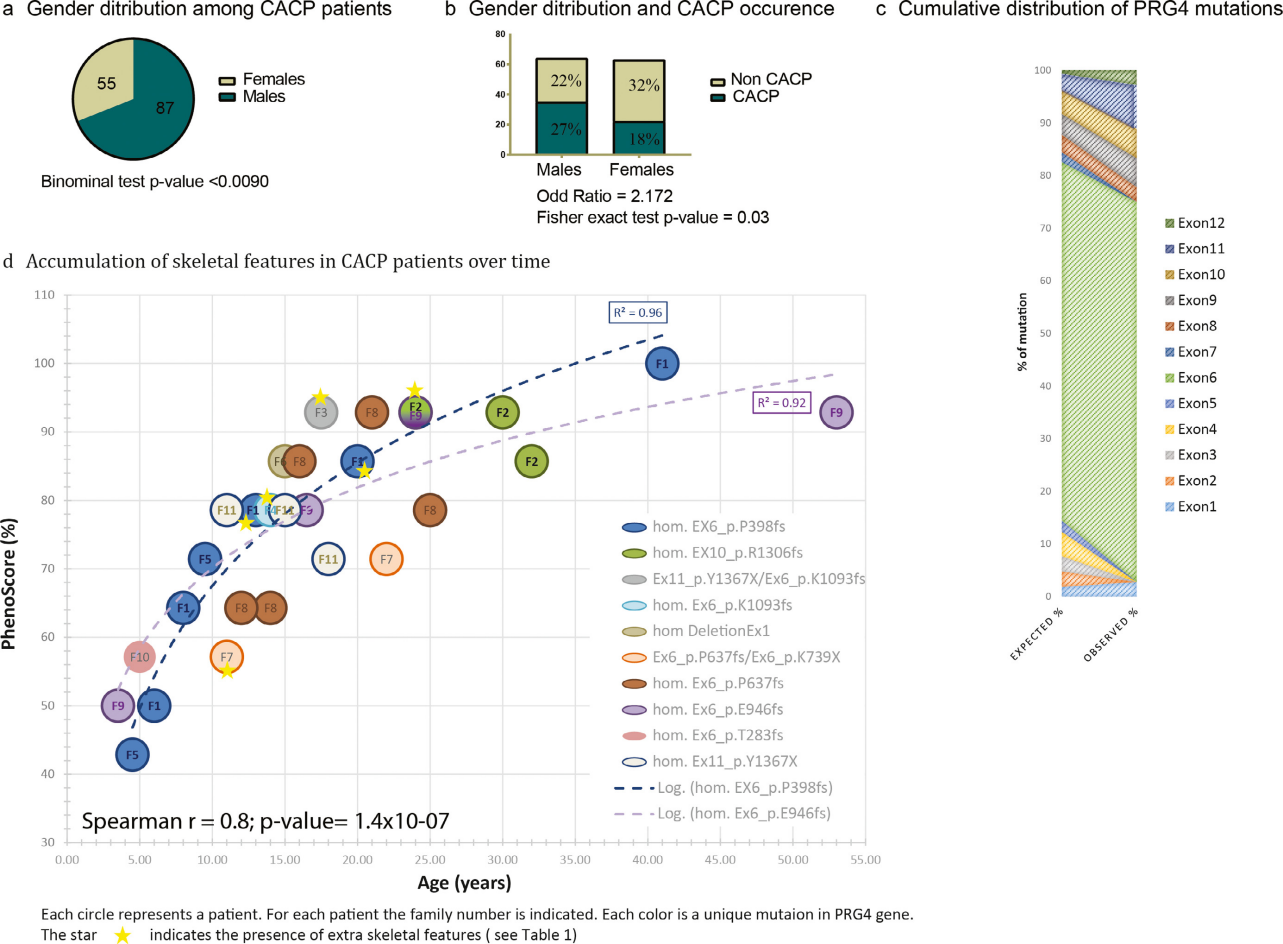
Correlations of *PRG4* mutations and clinical features. (a) Binominal test used to determine gender bias compared to a theoretical ratio of 1:1 gave a significant *p*‐value. Total number of published cases of CACP patients were used. (b) Fisher's exact test was used to determine gender bias linked to CACP disease. (c) Cumulative distribution of mutations across the coding region of the *PRG4* gene. The expected and observed percentages were calculated according to the sequence length (see Appendix S2). (d) Correlation between Phenoscore and age of the patient. Each circle represents a patient. For each patient, the family number is indicated in the circle. The star indicates the presence of extraskeletal features like pericarditis, ascites, pleurites, MVP (mitral valve prolapses), or MR (mitral regurgitation). The graph shows a significant correlation between the aging process and the severity of CACP (Spearman *r* = 0.8614, *p* = 3.23 × 10^−08^)

To look for a correlation between the clinical features and genetic data, we plotted the Phenoscore versus the age of the patient at the time of the study. The increase in age correlated significantly with the increased number of clinical findings (Spearman *r* = .8, *p* = 1.4 × 10^−07^). We reviewed the literature and found 65 males and 41 females with CACP (male to female gender ratio = 1.6, binominal test *p* = .025) (Akawi, Ali, & Al‐Gazali, 2012; Alazami, Al‐Mayouf, Wyngaard, & Meyer, 2006; Albuhairan & Al‐Mayouf, 2013; Bahabri et al., 1998; Basit et al., 2011; Ciullini Mannurita et al., 2014; Faivre et al., 2000; Peters et al., 2016)(Appendix S2c). In our cohort, we report 20 males with CACP and 15 females (males to female ratio = 1.3, binominal test *p* = .2). There is a significant male gender ratio bias in CACP population when we consider all reported cases including the present study (male to female ratio = 1.6, binomial test *p* = .009) (Figure 3a).

We next reviewed 18 publications that contained families for which a complete pedigree information was available, combining 9 unrelated families (49 subjects) from our study and 9 unrelated families (78 subjects) from previous reports (Appendix S2c). We found that male CACP patients were highly likely to have another brother with CACP (OR = 2.172; 95% confidence interval of 1.1–4.2; Fisher's exact test *p* = .03) (Figure 3b). Moreover, because of the autosomal recessive mode of inheritance of CACP, we expect 25% of the children (12.5 males, 12.5 females) to be homozygous for the mutated allele, while the remaining 75% would be either heterozygous carrier or homozygous wild type. We observed that 27% of the subjects are CACP males and 18% are CACP females.

## 4 DISCUSSION

CACP is a rare autosomal recessive inheritance disorder previously associated with alterations in the gene *PRG4*, coding for lubricin (Marcelino et al., 1999). Seven publications containing genetic data from CACP families have been published. With this study, our goal was to investigate the intra‐ and interfamilial clinical variability reported in CACP patients. To this end, we have investigated the DNA variations and clinical features of the largest cohort of CACP patients described so far and performed a complete review of the literature. We compared the clinical features of our patients to those reported in the literature. We then analyzed our cohort clinical features along with the genomic data to look for potential correlations.

### 4.1 Comparison to published cases

Ten of the families presented in this study are from southeast region of Turkey where rates of consanguineous marriage are high, and nine of the eleven families are reported to be consanguineous. Likewise, most of the previous published cases are from countries such as Saudi Arabia, United Arab Emirates, Egypt, and Pakistan (Akawi et al., 2012; Alazami et al., 2006; Albuhairan & Al‐Mayouf, 2013) where consanguinity rates are high. We calculated the male to female ratio of CACP population in a total of 29 unrelated families (including 18 previous reports) and found a significant male bias in the CACP population (male to female gender ratio = 1.6; binominal test *p* = .009). The total number of males (65) and females (66) in these families are similar, therefore the male bias seems not to drive higher mortality. On the other hand, the number of males with CACP is higher than expected for an autosomal recessive inheritance, while the number of females with CACP is similar to the expected number (Appendix S2c). It is important to take this observation with caution. Indeed, the Fisher's exact test *p*‐value is significant but modest. Further studies with pedigree information and complete genetic screening are necessary to confirm that CACP disease is over represented in male gender. Indeed, for most of the studies we have reviewed, the *PRG4* mutation status is unknown for patients as well as healthy members of the families.

In 1986, Bulutlar, Yazici, Ozdogan, and Schreuder (1986), who were among the first to describe CACP as a new syndrome, indicated that CACP can easily be misdiagnosed as JIA. Rheumatologic disorders are suspected in individuals with CACP because of a slow decrease in range of motion affecting large and small joints and increasing pain in the hip joints. Sixteen of our patients presented were referred for genetic evaluation with the initial diagnosis of JIA. Unfortunately, this misdiagnosis leads to a delayed age of diagnosis (12 years’ old for our cohort). Indeed, only 17% (6 of 35) of the patients presented here were diagnosed before 5 years of age and most of these patients were siblings of patients who had previously received a CCAP diagnosis. In 2004, Offiah, Woo, Prieur, Hasson, and Hall (2005) suggested considering CACP syndrome diagnosis for all patients that presented with noninflammatory arthropathy or atypical JIA.

Previous reports presented camptodactyly of the hands as the first symptom appearing during the first weeks or months of life, while other articular manifestations developed later and during the first 12 months (Alazami et al., 2006; Basit et al., 2011; Faivre et al., 2000; Offiah et al., 2005). For 68% of patients (19 of 28), camptodactyly was the earliest symptom and appeared to be mostly bilateral and progressive. Previous works has reported the wrists as the first large joints affected in early childhood period (Alazami et al., 2006; Faivre et al., 2000) which was replicated in our cohort. Large joint involvement was found in all of our patients and it included symmetrical noninflammatory arthropathy resulting in swelling, limited motion, and in flexion contractures. While all of the patients had wrists, elbows, and knees joints affected, ankle joints were affected only in some patients. Radiological findings of previously reported cases showed osteoporosis, increased joint space, small iliac wings, enlarged femoral head with short femoral neck, and coxa vara (Alazami et al., 2006; Basit et al., 2011; Faivre et al., 2000; Offiah et al., 2005). The report of coxa vara varies with studies reporting figures between 50% and 90% of CACP patients. All of patients in our study presented coxa vara and broad and short femoral neck as most distinct radiological findings. Besides osteoporosis, flat and enlarged femoral head, irregular acetabulum, small iliac wings, and intraosseous cysts were present in some patients (Figure 1b). Pericardial effusions were previously reported between 6% and 30% of CACP patients (Nandagopalan, Phadke, Dalal, & Ranganath, 2014). Although pericarditis and pleuritis were not observed in any of the follow‐up patients of family 1, an affected sister of one of the patients in this family died due to cardiac problems at 34 years of age. In contrast, two patients from family 2 and one patient from family 3 had pericarditis. In addition, four patients had MVP and MR on echocardiography (Table 1).

### 4.2 Genotype and phenotype analyses

In this cohort, our molecular screening identified six frame shift mutations, two nonsense mutations, and the first case of homozygous deletion of exon 1. Among the 27 mutations reported in the literature since 1993 (Marcelino et al., 1999), there are 15 are frame shift mutations, 4 stop codons, and 1 splice site acceptor (Appendix S2a). Our study brings the total number of disease‐causing mutations from 25 to 38. We show that 69% (9/13) of the mutations are in exon 6, while 4 mutations are found each in exon 1, 10, and 11. To this date, 26 (70%) mutations have been identified on exon 6, while the rest are distributed on the remaining part of the cDNA. There have not been any cases of CACP patients with mutations on exons 2, 3, 4, 5, or 7. All the 37 mutations reported so far in the literature are in the coding region shared by the five alternative transcripts. Indeed, exons 2, 4, and 5 are subject to alternative splicing, and mutation in these exons would leave the transcripts A and B intact (Appendix S2b). Therefore, deleterious mutations in exon 2, 4, or 5 would either lead to a different phenotype or have no deleterious consequences. The high number of mutations observed so far on exon 6 does not seem to represent a mutation hotspot with the number of cases published so far (binominal test *p* = .86). However, there seems to be a significant difference between the number of mutations in the regions not involved in alternative splicing (binominal test *p* = .046). This would support the idea that CACP appears when there is not any functional PRG4 protein left. All CCAP patients described in our study carried deleterious mutations predicted to abolish the functions of both copies of PRG4 protein (material and methods translate tool).

Based on CACP mutation profiles, several authors previously stipulated that the syndrome is due to a complete lack of the protein PRG4 (Alazami et al., 2006; Basit et al., 2011; Ciullini Mannurita et al., 2014). In addition, studies using an antibody against the C‐terminal and N‐terminal of PRG4 protein showed its absence in CACP patient's synovial fluid, while it was detected in samples patients with rheumatoid arthritis and osteoarthritis (Ai et al., 2015). It has also been shown that the synovial fluid from patients with CACP lack lubricating properties (Jay et al., 2007). Studies revealing the functions of different PRG4 domains have emerged recently. PRG4 core 1 O‐glycosylation has been suggested to carry lubricating functions, while core 2 structures have been identified as the oligosaccharides precursors of inflammation epitopes. Indeed, the glycol epitopes on lubricin have the potential of strong interaction with selectin, galectins, and potentially other glycol‐binding proteins to facilitate inflammation (Ali et al., 2014; Jay, 1992; Jay, Harris, & Cha, 2001), however CACP patients do not present any signs of inflammation. One CACP family with a dinucleotide transversion (4190CC→AG) creating a nonsense codon on the last exon has been reported (Marcelino et al., 1999). The nonsense‐mediated mRNA decay (NMD) system termination does not degrade abnormal mRNAs if the mutation is in the last exon of the gene or if the mutation is within the last 50 bp from the last exon–intron junction of the gene (Brogna & Wen, 2009; Perrin‐Vidoz, Sinilnikova, Stoppa‐Lyonnet, Lenoir, & Mazoyer, 2002). In vitro experiments have shown that this mutated protein does not undergo the normal process of SPC‐mediated cleavage within the PEX domain (Rhee et al., 2005a), meaning that the PRG4 protein is nonfunctional. Indeed, full‐length protein presents an optimal lubricating function when the intact negatively charges STP‐rich region and positively charged at the N‐ and C‐terminal regions are intact (Ali et al., 2014; Lee, Muller, Rezwan, & Spencer, 2005; Swann, Hendren, Radin, Sotman, & Duda, 1981). In our cohort, we report three siblings with homozygous p.Y1367X (c.4101C>G) mutation predicted to escape the NMD. Indeed, the mutation is 17 bp from the last exon–intron junction. A recent study has reported that the NMD efficiency is variable between individuals and that difference of efficiency could explain some interindividual variabilities in phenotypes (Nguyen, Wilkinson, & Gecz, 2014). Interestingly, in mice the efficiency of NMD has been proven to vary among different tissue (Zetoune et al., 2008). Finally, recent studies have demonstrated that some transcripts escape the NMD system producing a truncated protein. For example, PTCs that are unable to trigger NMD cause dominantly inherited forms of, for example, β‐thalassemia (Bhuvanagiri, Schlitter, Hentze, & Kulozik, 2010; Thein et al., 1990). Unfortunately, it is currently not possible to predict which mRNA will trigger or escape NMD based on the sequence features only (Karousis, Nasif, & Muhlemann, 2016). It is therefore required to perform a case per case study to see the consequence of each mutation. Future studies aiming to study (NMD) system efficiently on CACP patients’ samples could shed lights on the effect of these PTC mutations of *PRG4* gene in CACP patients’ phenotype.

CACP has been described as a clinically variable but genetically homogenous disease (Faivre et al., 2000), and the disease inter‐ and intravariability had been repetitively described by authors, without any emerging consensus on the origins of such variability.

### 4.3 Genotype and phenotype analyses

While the first symptoms of CCAP seem to appear early, the disease becomes more severe with time (Figure 3d). As expected, we found a significant correlation between the age of the patient and the number of clinical features (Phenoscore) (Spearman *r* = .86, *p* = 3.23 × 10^−08^). This increase in symptoms is likely due to cumulative mechanical stress over time (Jahn, Seror, & Klein, 2016; Lorenz & Richter, 2006). Differences in mechanical stress may also explain at least part of the intrafamilial variability (Figure 3d). Indeed, the long‐term follow‐up of CACP patients reveals hip and spine involvement in some cases. We also observed that severe hip joints involvement developed in patients older than 10 years. Indeed, older patients described an increase in their pain after reaching 10 years old. This corresponds to the age when large joint contractures increase. Patient 1 from family 1 (followed since 5 years of age) developed joint pain at the age of 9, which gradually increased. Patient 5, who is 41 years old at the time of this study, developed severe hip involvement at age 25, and required a hip prosthesis operation at the age of 35. We observed significantly increased lumbar lordosis only in these older patients (Figure 1b). However, the first reported symptoms among individuals within the same family remain variable (Faivre et al., 2000). For example, family 1 reported camptodactyly of hands at 5–6 months of age as the earliest symptom for most of the patients. On contrary, for patient 4 from the same clan, wrist involvement was reported to be the first symptom, while camptodactyly appeared together with elbow and knee involvement 1 year later.

The first symptoms are also variable between families. Previous reports presented camptodactyly of the hands as the first symptom appearing during the first weeks or months of life, while other articular manifestations developed later and during the first 12 months (Alazami et al., 2006; Basit et al., 2011; Faivre et al., 2000; Offiah et al., 2005). For 68% (17/25) of our patients, camptodactyly of the hands was the earliest symptom.

For a similar age, the nature and severity of skeletal features are also variable. Indeed, in family 2, even though the hip involvement was mild, we also observed knee, ankle, elbow, and shoulder joints involvement. For the same age, the skeletal findings were more numerous, but milder in family 2 compared to family 1. At 17.5 years old, the patient carrying a p.Y1367X/p.K1093fs mutation (family 3, patient16) presented the highest number of skeletal findings, and more importantly, an accumulation of severe extraskeletal features such as pericarditis, untreatable ascites, and pleuritis. We observe that for family 1 the accumulation of skeletal features was proportional to the age of the patient (logarithmic regression). For other families (e.g., Family 8) there was more variability in Phenoscore, even though a positive correlation existed (Figure 3d). It is worth noting that the patient carrying the exon 1 mutation (patient 20, family 6) did not present a higher Phenoscore than other patients nor did the patient present any sign of extraskeletal features. The patient did not present any visceral problem, yet at 15 years of age, the skeletal findings were severe and the patient reported pain earlier than other patients.

**Figure 4:**
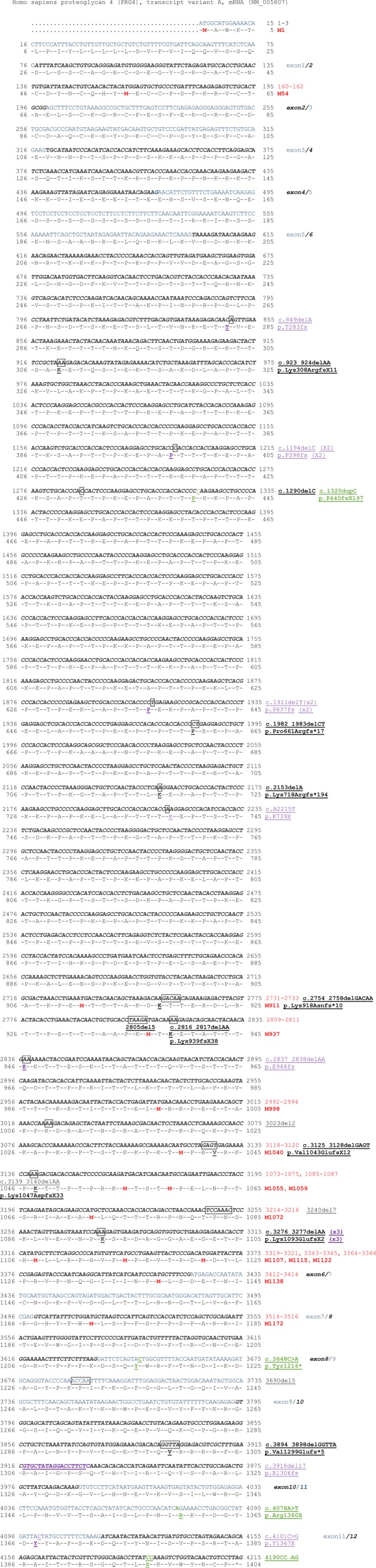
*Homo sapiens* proteoglycan 4 (*PRG4*) transcript variant A, mRNA (NM_005807) annotated cDNA sequence. Alternative exons are depicted on the cDNA sequence with alternative black and blue color (e.g., exon 1/2). Start codons are identified in red at the cDNA (160–162) and protein level (M54). All the reported mutations are displayed at cDNA and/or protein level. Previously reported deletions and insertions (3690del5) and point mutations (c.3648C≥A) and mutations discovered in the present study (c.3918del17)

β‐Globin transcript with nonsense mutations in the first exon are known to escape the NMD producing a dominant negative form of the disease (Neu‐Yilik et al., 2011). In a similar observation, nonsense mutation in exon 1 (p.Tyr14*) of paired mesoderm homeobox protein 2B (*PHOX2B*, OMIM: 603851) leads to an N‐terminal truncated protein via translational reinitiation at either p.Met18 or p.Met21 also located in exon 1 (Cain et al., 2017; Trochet et al., 2009). In addition to the first methionine codon, PRG4 contains Met55 on exon 2, 13 methionine codons on exon 6, and 1 in exon 8 (Figure 4).

We observed several extraclinical features present in CACP patients (indicated with a star in Fig 3d). Approximately 20% of patients with CACP also had pericarditis, which was not associated with age, gender, or mutation type or localization. Interestingly, a recent study found that PRG4 protein is also abundantly present in the pericardium, with a modified post‐transcriptional form than it is in the synovial fluid (Ikegawa et al., 2000). This supports the idea that PRG4 protein is important for pericardium. However, a mouse knockout model *prg4* ^(−/−)^ shows no signs of pericardial overgrowth (Rhee et al., 2005b).

Additional genomic variations could account for the interindividual and interfamilial phenotypic variabilities observed in CACP patients. The presence of additional variants could fine tune the traits produced by the malfunction of PRG4 protein. Indeed, 80% of the families are consanguineous, and the probability of accumulating homozygous genomic aberrations is therefore higher than in the general population. Additionally, CACP syndrome shows characteristics of oligogenic inheritance. For example, cystic fibrosis is an example of an autosomal recessive disease showing a very complex association between genotype and clinical phenotype. Indeed, it is not possible to predict individual outcome based on cystic fibrosis transmembrane regulator gene (*CFTR*; OMIM: 602421) genotype only. The expression of the disease is influenced by various factors that make phenotype variability extend along a wide spectrum (Castellani & Assael, 2017). In an extreme example of phenotypic variation, males can manifest bilateral agenesis of the vas deferens (CBAVD) with no digestive or respiratory involvement (Bombieri et al., 2011).

Among the patients in our cohort with exome sequencing information, we searched for co‐concurrent mutations in genes that may be linked to PRG4 or its pathways (Appendix S3). More specifically, we looked for mutations in hyaluronan synthase 1 (*HAS1*; OMIM: 601463) and aggrecan (*ACAN*; OMIM: 155760); the main and ubiquitous constituents of synovial fluid and cartilages along with *PRG4* (Jahn et al., 2016). For example, the deficiency of either of hyaluronan synthase 1 and *PRG4* or the dysfunction of *PRG4* appears to be detrimental to the lubricating function of the synovial fluid (Ludwig, Hunter, & Schmidt, 2015). With limited number of data and samples we present here, it is not possible to draw any conclusion about secondary mutations and our analyses are purely exploratory by nature. The investigations are summarized in Appendix S3.

## 5 CONCLUSION

With this study, we contribute to the catalog of CACP causing variants. We prove that CACP is a disorder that effects large and small joints, progress with the age of the patient, and shows intra‐ and interfamilial clinical variations. The main component of intrafamilial variability is the patient's age, probably reflecting the accumulation of mechanical ware. Because the severity of the disease is dependent on the patient's age, we suggest reporting the patient's age at the time of the study when assessing CACP clinical features. Our data support the idea that CACP appears when both copies of *PRG4* are dysfunctional. However, our results indicate that the total absence of PRG4 protein is not required to lead to CACP. Indeed, one case of a CACP family with a homozygous nonsense mutation in *PRG4* gene was predicted to escape NMD. There have been numerous examples showing that there is a continuum between purely Mendelian monogenic disease and complex traits (Badano & Katsanis, 2002), and in CACP the *PRG4* locus contribute to the majority of the phenotype. In addition, the interfamilial variabilities as well as CACP's nonskeletal features do not seem to correlate with age, gender, ethnicity, and geographic localization. We propose that CACP is an oligogenic disorder with at least an additional locus explaining the interfamilial variabilities. Larger cohorts with extensive clinical data and exome sequencing methods could elucidate the interfamilial clinical variability. We believe that this report will increase awareness of this familial arthropathy condition and the characteristic clinical and radiological findings will facilitate the differentiation from the common childhood rheumatic diseases such as JIA.

## CONFLICT OF INTEREST

The authors have no conflict of interest to declare.
